# Supplementary material for: Bile Acids Activate NLRP3 Inflammasome, Promoting Murine Liver Inflammation or Fibrosis in a Cell Type-Specific Manner
Source: Cells. 2021 Oct 1;10(10):2618. doi: 10.3390/cells10102618 (PMC8534222; doi:10.3390/cells10102618)
Supplement: Supplementary file 1 [file cells-10-02618-s001.zip › cells-1349572-supplementary.pdf]

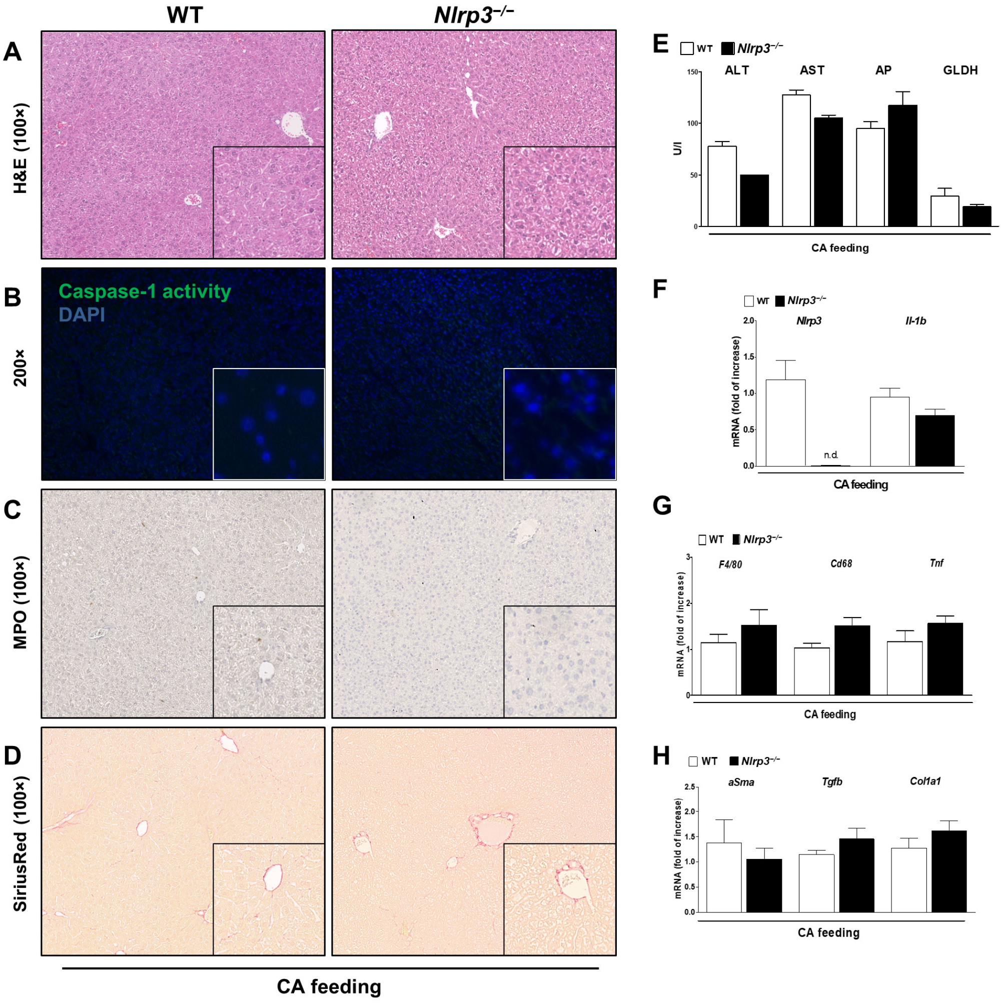

Figure S1: feeding with CA did no harm to neither WT nor Nlrp3$^{-/-}$ mice

H&E staining (A) showed no bile infarcts or necrotic areas in liver sections after feeding of CA to WT or Nlrp3$^{-/-}$, so no change of Transaminases was observed in Nlrp3$^{-/-}$ mice when compared to WT (E). Moreover, mRNA levels of Il-1b (F) and Caspase-1 activity (B) remained the same in Nlrp3$^{-/-}$ mice compared to WT. No invasion of MPO-positive immune cells was observed (C) and mRNA markers for immune cell invasion (F4/80, Cd68,) and proinflammatory macrophages (Tnf) (G) did not alter in Nlrp3$^{-/-}$ whole liver lysate when compared to WT. Liver sections of WT and Nlrp3$^{-/-}$ developed no fibrosis (D) and mRNA levels of fibrotic markers (H) did not differ in WT and Nlrp3$^{-/-}$.

n=6

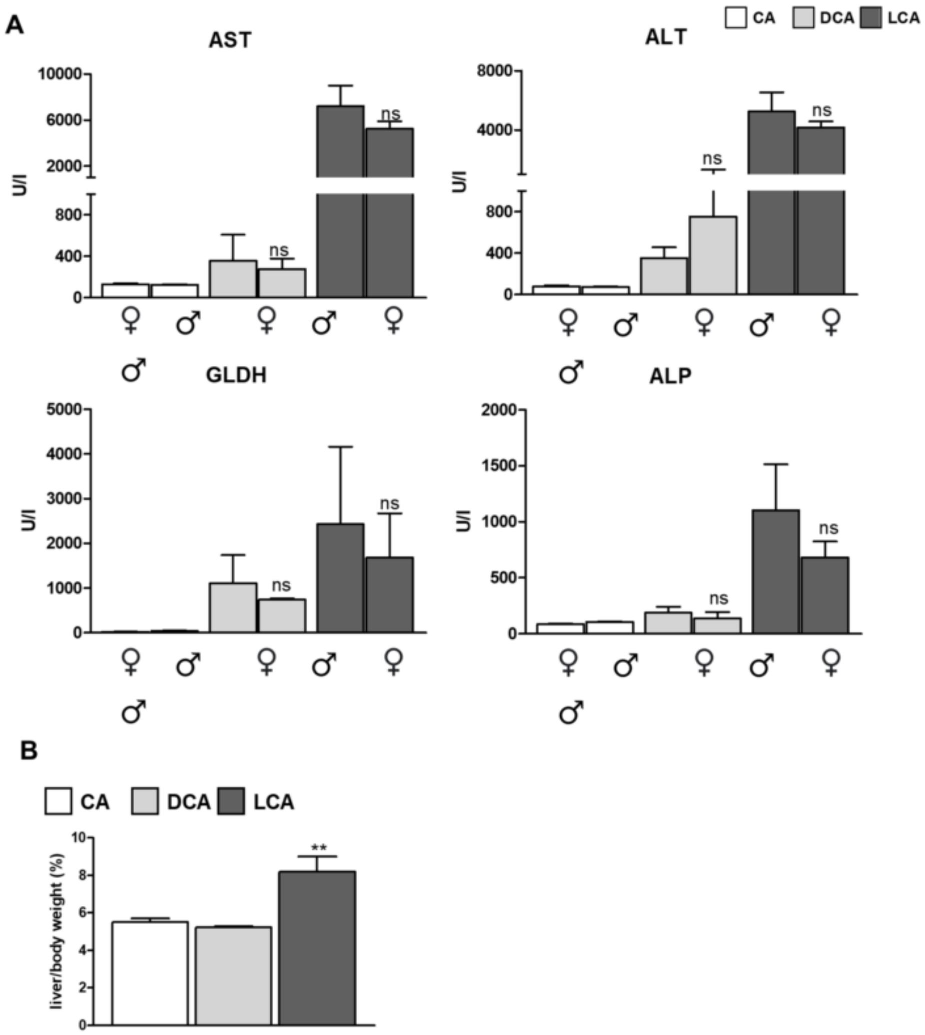

Figure S2: Levels of ALT, AST, ALP and GLDH did not differ between male and female mice after feeding with CA, DCA or LCA acid (A). Liver-body-weight ratio was increased after LCA feeding in contrast to CA or DCA feeding.

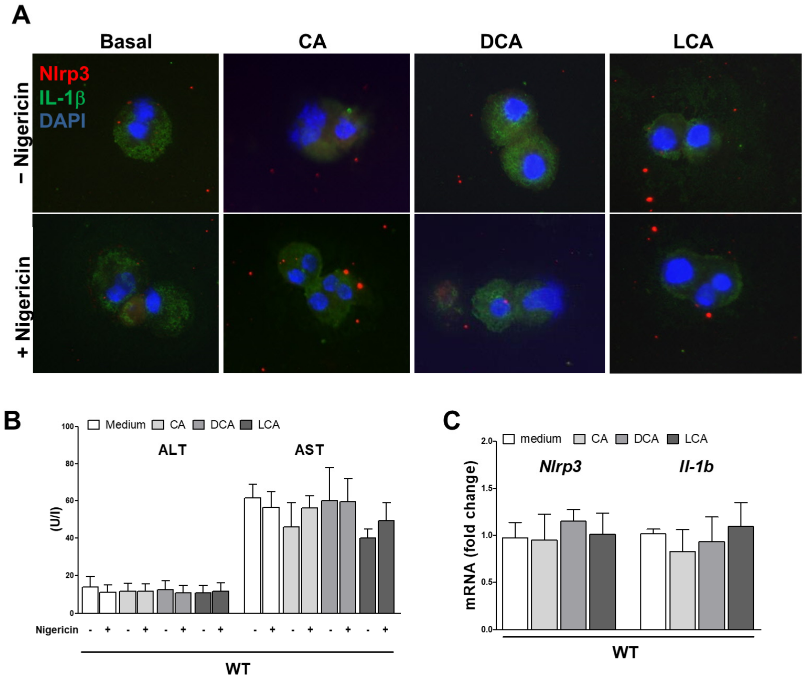

Figure S3: Hepatocytes did not react with NLRP3 activation nor cell damage to exposure with CA, DCA or LCA

Stimulation of primary hepatocytes with CA, DCA or LCA did not harm the hepatocytes as indicated by constant Transaminase levels in the supernatant and morphologically intact cells (B, A). No upregulation of NLRP3 or IL-1β was observed on protein or mRNA level (A, C).

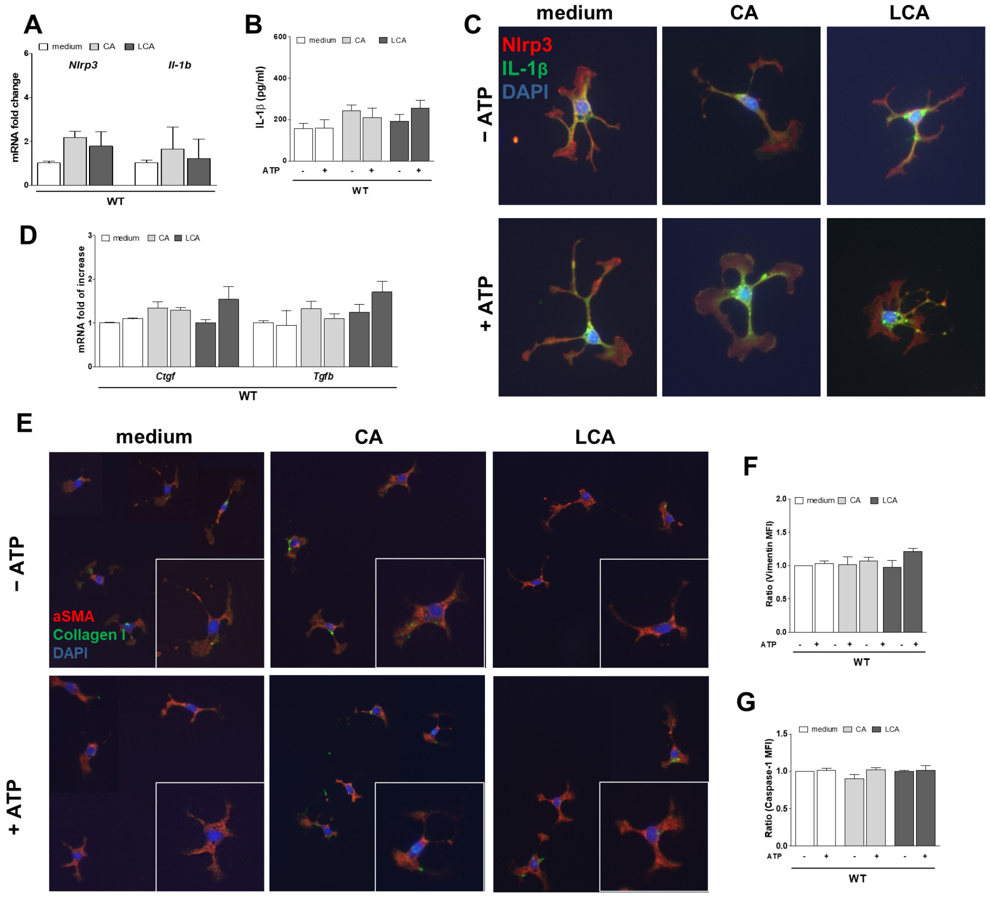

Figure S4: LCA and CA could not activate the NLRP3 inflammasome in HSC and led to no fibrotic phenotype

After exposure to LCA or CA no activation of NLRP3 inflammasome was triggered in HSC as basal mRNA and protein levels of NLRP3 and IL-1β, and basal Caspase-1 activity remained (A, B, C, G). Furthermore, no morphological change nor increased deposition of Collagen I or Vimentin was observed (E, F). Fibrotic mRNA markers (*Ctgf, Tgfb*) showed no significant upregulation in HSC after stimulation with LCA or CA (D).

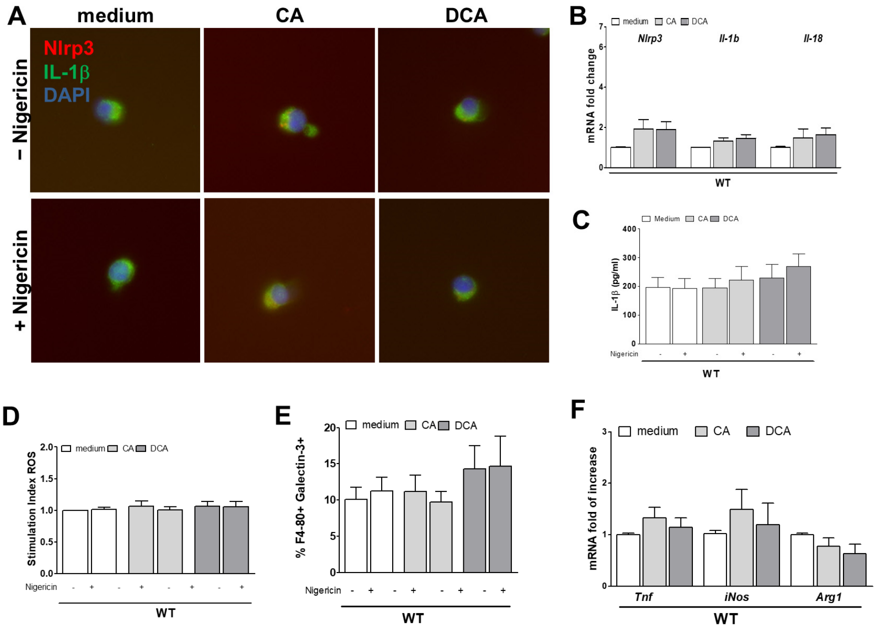

Figure S5: DCA and CA could not activate the NLRP3 inflammasome in KC and led to no inflammatory phenotype

KC did not activate the NLRP3 inflammasome after exposure with DCA or CA as shown by stable mRNA levels of *Nlrp3, Il-1b* and *Il-18* (B) and no increase in protein levels of NLRP3 and IL-1β (A, C). No oxidative stress was induced by stimulation with DCA or CA as indicated by stable ROS production (D). Pro- and antiinflammtory mRNA markers (*Tnf, iNos, Arg1*) (F) and percentage of Galectin 3-positive cells (E) showed no changes after addition of DCA or CA.
